# Delayed laparoscopic peritoneal washout in non-operative management of blunt abdominal trauma: a scoping review

**DOI:** 10.1186/s13017-022-00441-z

**Published:** 2022-07-02

**Authors:** Megan Chu, Nathan How, Alysha Laviolette, Monika Bilic, Jennifer Tang, Maham Khalid, Cecily Bos, Timothy J. Rice, Paul T. Engels

**Affiliations:** 1 Division of General Surgery, McMaster University, Hamilton, Canada; 2 Michael G. DeGroote School of Medicine, McMaster University, Hamilton, Canada; 3 Temerty Faculty of Medicine, University of Toronto, Toronto, Canada; 4 Trauma Program, Sunnybrook Health Sciences Centre, Toronto, Canada; 5 Trauma Program, Hamilton General Hospital, Hamilton Health Sciences, Hamilton, Canada

**Keywords:** Delayed laparoscopy, Blunt abdominal trauma, Peritoneal washout

## Abstract

**Objectives:**

Non-operative management (NOM) of blunt abdominal trauma has become increasingly common in hemodynamically stable patients. There are known complications of NOM from undrained intra-abdominal fluid accumulations including hemorrhage and peritonitis that require delayed operation. Thus, delayed operation can be considered as part of the overall management plan, instead of failure, of NOM. The aim of this scoping review is to establish key concepts regarding delayed laparoscopic peritoneal washout (DLPW) following NOM of blunt abdominal trauma patients.

**Methods:**

MEDLINE, EMBASE, CENTRAL, and gray literature were systematically searched. Studies were included if they investigated or reported on the use of delayed laparoscopy involving peritoneal washout following NOM of blunt abdominal trauma patients. Bibliographies of included studies were manually reviewed to identify additional articles for inclusion.

**Results:**

From 910 citations, 28 studies met inclusion criteria. This included seven case reports, eleven case series or observational cohort studies, six review articles, two management guidelines, one textbook chapter, and one randomized clinical trial. For those reported, medium grade liver injuries proved most common (95.2%). Indications for DLPW were primarily clinical features and changes in imaging findings, highlighting the importance of close observation. Authors reported clinical improvement after DLPW regarding symptomatology, vital signs, and biochemistry. A relatively high transfusion demand was reported with a mean of four units of packed red blood cells pre-operatively. Length of stay and post-operative complications were consistent with previously reported experiences with blunt abdominal injuries.

**Conclusions:**

DLPW is beneficial in blunt abdominal trauma patients following NOM with improvement in symptoms, SIRS features, and a possible reduction in hospital length of stay. This study is limited by low-quality evidence and skewing of data toward isolated hepatic injuries. Future prospective cohort study comparing NOM with and without DLPW is required.

## Introduction

Non-operative management (NOM) of blunt abdominal trauma has become acknowledged as a safe treatment option in many injury patterns for hemodynamically stable patients and is now considered to be part of the standard of care [1, 2]. To date, the liver is the most commonly injured organ in blunt abdominal traumas, and more than 80% of patients are treated without the need for operative intervention [2, 3]. Delayed operation has typically been considered a failure of non-operative management. However, as argued by Letoublon et al. it is increasingly being considered as part of the overall management plan due to complications associated with NOM that are known to require delayed operative intervention [2, 4, 5]. Such intervention may include delayed laparoscopic peritoneal washout (DLPW).

Prior to NOM becoming the standard of care, drainage of blood and fluid collections from the peritoneum was performed at the time of initial operative management for blunt trauma [5, 6]. As a result of the movement toward non-operative management, patients who do not receive surgical treatment and concurrent drainage are thus at a greater risk for intra-abdominal fluid accumulations [4, 5]. These undrained collections may lead to or obfuscate complications such as hemorrhage, biliperitoneum, peritonitis, abdominal compartment syndrome, systemic inflammatory response and/or respiratory distress [2–5]. Chemical peritonitis from bile or blood causes significant pain and a physiologic ileus, delaying enteral nutrition and physical rehabilitation. The associated pain may be distracting and create diagnostic uncertainty during serial abdominal examinations. Additionally, abdominal hematomas are at risk for secondary infection [7], particularly in the pro-inflammatory trauma state which promotes intestinal bacterial translocation [8]. As a result, these factors may increase morbidity, prolong patient suffering, and result in delayed patient recovery post-trauma [9].

To date, there has been considerable research investigating delayed operative intervention in patients originally treated with NOM for blunt abdominal trauma [2, 4, 5]. Notably, there currently remains a paucity of information regarding the indications for and outcomes from delayed laparoscopy specifically for the purpose of peritoneal washout. This scoping review aims to establish key concepts regarding delayed laparoscopy for the purpose of peritoneal washout following blunt abdominal trauma and identify gaps in the literature to guide future research.

## Methods

Where applicable, Preferred Reporting Items for Systematic Reviews and Meta-analyses extension for scoping reviews (PRISMA-ScR) recommendations were integrated into our methodology. No protocol was registered prior to initiation of the study.

### Research question

What are the pre-operative clinical course and post-operative outcomes of patients undergoing delayed laparoscopic washout in blunt abdominal trauma?

### Search strategy

**Fig. 1:**
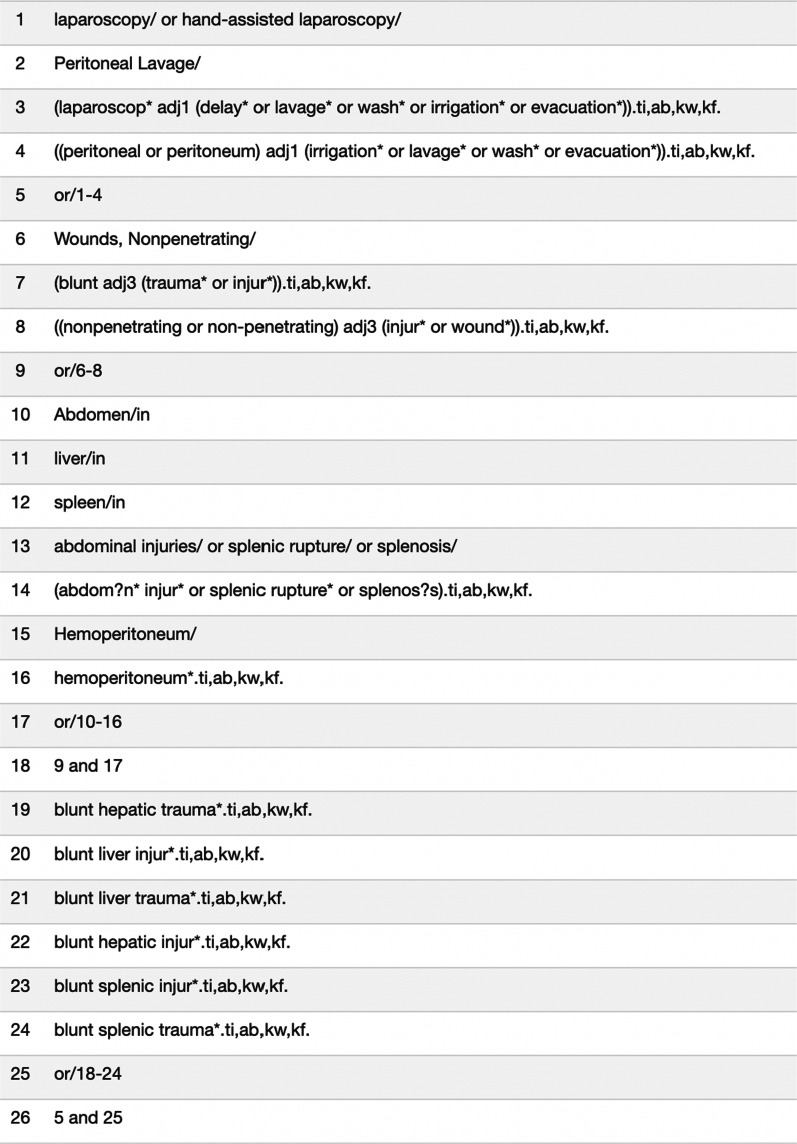
Sample explicit search strategy used for MEDLINE database

A comprehensive search of the following databases from 1986 to September 2020 was conducted: MEDLINE, EMBASE, Cochrane Central Registrar of Controlled Trials (CENTRAL), and the major clinical trial registries (ClinicalTrials.gov: http://clinicaltrials.gov/; International Clinical Trials Registry Platform Search Portal (ICTRP: https://apps.who.int/trialsearch/) for ongoing trials. The explicit search strategy for the MEDLINE database is illustrated in Fig. 1. The gray literature was further interrogated using New York Academy of Medicine’s Gray Literature Report, Canadian Agency for Drugs and Technologies in Health, Open Grey, and Canadian Institute for Health Information.

The study investigators worked alongside a medical research librarian to design and conduct a search strategy. A review protocol was not registered prior to starting the study. To ensure assessment of all relevant articles, references of published studies were searched in addition to gray literature (e.g., conference abstracts, unpublished trial data, presentations). There were no language restrictions. Additional studies were identified for inclusion through manual review of bibliographies of selected studies.

### Eligibility criteria

Studies were eligible for inclusion if they investigated or reported on the use of delayed laparoscopy involving peritoneal washout following non-operative management of patients sustaining blunt abdominal trauma. Delayed laparoscopy was defined as greater than 24 h between initial injury and laparoscopy. We included case studies, cohort studies (prospective or retrospective), randomized controlled trials and reviews. Our exclusion criteria were: 1. patients who sustained penetrating trauma; 2. those who were managed initially with operative treatment; 3. patients who did not receive laparoscopy following NOM, 4. cases involving additional surgical procedures other than washout at the time of laparoscopy.

### Outcomes

The primary outcome of interest is to investigate the clinical course preceding DLPW including patient demographics, mechanism of injury, pre-operative interventions, and operative indications, in addition to patient outcomes following NOM for blunt abdominal trauma. This includes initial presenting injuries, delay between initial injury and laparoscopy, indications for laparoscopy, operative findings, morbidity and mortality, post-operative interventions, and pre-operative transfusion requirements.

### Study selection procedure

**Fig. 2:**
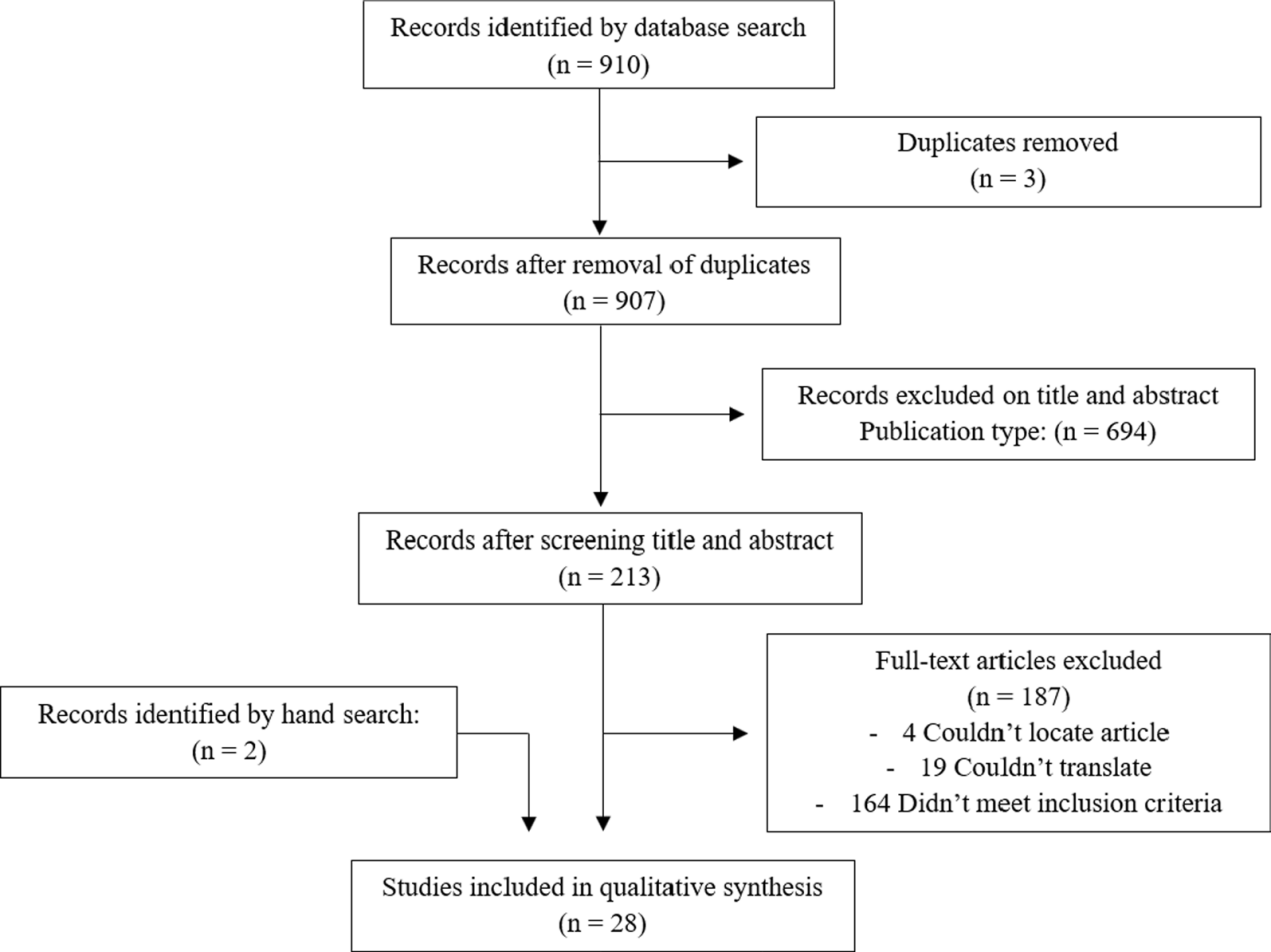
PRISMA Flowchart diagram illustrating study selection process

Eligible articles were identified through two phases. In phase 1, five reviewers (M.C., M.B., A.L., J.T., and M.K.) independently evaluated the titles and abstracts of the retrieved publications and removed irrelevant articles. In the second phase, the full texts of the remaining articles were reviewed by four reviewers (M.C., M.B., A.L., J.T.) using the aforementioned inclusion criteria. A PRISMA flow diagram illustrating this process can be found in Fig. 2. Any discrepancy about study inclusion and exclusion between two reviewers was resolved by a third reviewer (N.H.). Throughout this process, reviewers were not blinded to authors, institutions, or the journal where the manuscript was published.

### Data abstraction

Two reviewers (M.C., N.H.) independently conducted data extraction onto a data collection tool designed a priori*.* Extracted data included study characteristics (e.g., author, year of publication, country), patient characteristics (e.g., age, gender, mechanism of injury, organ injury), and clinical course (e.g., non-operative management, indication for laparoscopy, type of peritoneal fluid, post-operative interventions, morbidity/mortality, etc.). For comparison purposes, data were converted to a common unit where possible.

### Quality assessment

The Newcastle–Ottawa Scale was used to assess risk of bias, completed by one reviewer. This scale is made up of three domains with a maximum of nine stars for each study: selection (up to four stars), comparability (up to two stars), and outcomes (up to three stars). The Newcastle–Ottawa Scale was converted to AHRQ standards. Studies with 3 or 4 stars in selection domain, 1 or 2 stars in comparability domain, and 2 or 3 stars in outcome/exposure domain were deemed good quality. Studies with 2 stars in selection domain, 1 or 2 stars in comparability domain, and 2 or 3 stars in outcome/exposure domain were deemed fair quality. Finally, studies with 0 or 1 star in selection domain, or 0 stars in comparability domain, or 0 or 1 stars in outcome/exposure domain were deemed poor quality.

## Results

### Study selection

A total of 910 potentially relevant articles were identified. Duplicates were removed, which left 907 articles that met the inclusion criteria for title and abstract screening. Following the title and abstract screening, 694 articles were excluded, leaving 213 full-text articles to be reviewed. An additional 187 articles were excluded upon full-text review. Two additional articles were identified by hand through searching bibliographies of included studies, leaving a total of 28 included studies. Inter-rater agreement for inclusion criteria, as measured by Fleiss’ kappa was 0.91 (95% CI 0.819 to 1). Exclusion criteria included: laparoscopy solely used for diagnostic purposes (*n* = 33), no blunt abdominal trauma (*n* = 1), laparoscopy before trial of NOM (*n* = 39), surgical intervention during laparoscopy (*n* = 17), or no therapeutic laparoscopy following NOM (*n* = *74*).

### Study characteristics

Included research had a variety of study designs including one randomized clinical trial [10], ten observational cohort studies [2, 4, 5, 11–17], seven case reports [3, 18–23], six review articles [6, 24–28], two management guidelines [29, 30], and one textbook chapter [31]. The single clinical trial was only published in abstract format. They were published in eleven countries with a majority being from the USA and France. Language of original text was predominantly English (88.5%) with two articles in French and one article in Portuguese. Dates of publication ranged from 1993 to 2019 with the great majority being published after 1999.

### Patient demographics

In total, the included papers reported on 108 patients who underwent DLPW after initial non-operative management of blunt abdominal trauma. Of those for which it was reported, 68.9% were male (Table 1). Average reported age was 31.6 years with a range from 11 to 61.

**Table 1:** Patient demographics from included studies

Patient demographics	Total
*Sex*	
Male	22 (68.8%)
Female	10 (31.2%)
*Age (years)*	
Mean ± SD	31.6 ± 10.0
(Minimum, maximum)	11–61

### Types of trauma

All included patients suffered a blunt trauma mechanism. Motor vehicle collisions (MVCs), including motorbike accidents, accounted for most (68.4%) of the mechanisms followed by falls (15.8%), horse kicks (10.5%), and falling objects (5.3%). No cause was explicitly reported for 82.1% of patients meeting inclusion criteria; all of these included patients sustained known blunt abdominal trauma however, demographics were often grouped together with patients receiving immediate laparoscopy. Of those for which it was reported, organs injured included liver (95.2%), small bowel (3.2%), and pancreas (1.6%). Organ injury was unspecified in 40.6% of patients (Table 2).

**Table 2:** Trauma descriptors

Types of trauma	Total (%)
Mechanism of injury	
Motor vehicle collision	13 (68.4)
Falls	3 (15.8)
Horse kicks	2 (10.5)
Falling objects	1 (5.3)
Organ injured	
Liver	60 (95.2)
Grade II	3 (5)
Grade III	14 (23.3)
Grade IV	20 (33.3)
Grade V	4 (6.7)
Unspecified	19 (31.7)
Small bowel	2 (3.2)
Pancreas	1 (1.6)

### Pre-operative course

Non-operative management (NOM) for patient injuries consisted of observation for 86.5% and angiography with or without embolization for 12.7% of patients (Table 3). One patient (1%) presented to medical services in a delayed fashion. Indications for NOM was unknown for 3.8% of patients. All patients underwent an initial CT scan of the abdomen and pelvis. Following the initial assessment and admission, 35.8% of patients underwent repeat imaging or invasive investigations prior to laparoscopy, which included computerized axial tomography (CT), ultrasonography, endoscopic retrograde cholangiopancreatography (ERCP), and hepatobiliary imiodiactic acid (HIDA) scan in 27.4%, 4.7%, 1.9%, and 1.9% of patients, respectively. Additionally, 13.2% of patients received transfusion pre-laparoscopy, and those patients required on average 4.1 units of red blood cells (RBC) assuming 1 unit of RBC was 300 mL where volume was reported instead of units [32].

**Table 3:** Description of pre-operative course including management, investigations, and interventions

Pre-operative course	Total
*Non-operative management*	
Observation	87 (85.3%)
Angiography with or without embolization	13 (12.7%)
Bowel rest	1 (1%)
Delayed presentation	1 (1%)
*Investigations and interventions*	
Computerized axial tomography	29 (27.4%)
Ultrasonography	5 (4.7%)
Endoscopic retrograde cholangiopancreatography	2 (1.9%)
Hepatobiliary imiodiactic acid	2 (1.9%)
*Pre-laparoscopic transfusion*	
Mean ± SD	4.1 ± 3.1
(Minimum, maximum)	1–11.67

### Operative intervention

On average, operative intervention occurred on day 5 with a range of 2–35 days (Table 4). Reported indications for DLPW included sepsis/SIRS criteria (65%), imaging findings (54%), hemodynamic instability (23%), bloodwork results (20%), abdominal pain/peritonitis (15%), experimental arm in RCT (13%), pulmonary dysfunction (4%), intra-abdominal hypertension (4%), abdominal distension (4%), suspected infection collection (3%), ileus (2%), abdominal compartment syndrome (1%), decreased urine output (1%), and poor oral intake (1%). Percentages add up to more than 100% as authors often listed more than one reason for the decision to operate. The type of fluid identified at the time of OR was most often blood (55.9%), followed by bile (48.4%), blood and/or bile (23.7%), infected collection (3.2%), and lastly chyle (1.1%). The type of fluid was not reported for thirteen patients.

**Table 4:** Description of operative course including indications and findings

Operative intervention	Total
*Time to laparoscopy (days)*	
Mean ± SD	5.3 ± 4.7
(Minimum, maximum)	2–35
*Indications for laparoscopy*	
Sepsis/SIRS criteria	65 (65%)
Imaging findings	54 (54%)
Hemodynamic instability	23 (23%)
Bloodwork results	20 (20%)
Abdominal pain/peritonitis	15 (15%)
Intervention in RCT	13 (13%)
Pulmonary dysfunction	4 (4%)
Intra-abdominal hypertension	4 (4%)
Suspected infected collection	3 (3%)
Ileus	2 (2%)
Abdominal compartment syndrome	1 (1%)
Decreased urine output	1 (1%)
Poor oral intake	1 (1%)
Confirmation of leak cessation	1 (1%)
*Fluid type during laparoscopy*	
Blood	52 (55.9%)
Bile	45 (48.4%)
Blood and/or Bile	22 (23.7%)
Infected collection	3 (3.2%)
Chyle	1 (1.1%)

### Health related outcomes

Average hospital length of stay (LOS) was 14 days with a range of 11–43 days (Table 5). Post-operative interventions included surgical drains, ERCP with stenting, and IR drainage in 19.8%, 4.7%, and 3.8%, of patients, respectively, with patients commonly requiring more than one intervention. The single RCT reported an improvement in LOS from 8.93 days (± 2.89) in the control group to 5.69 days (± 1.887) in the DLPW group. No statistical test for significance of this difference was reported.

**Table 5:** Post-operative patient outcomes and interventions

Health related outcomes	Total
*Hospital length of stay (days)*	
Mean ± SD	14 ± 8.9
Minimum, maximum	11–43
*Post-operative interventions*	
Surgical drains	21 (19.8%)
ERCP with stenting	5 (4.7%)
IR drainage	4 (3.8%)
Antibiotics	2 (2.8%)
*Morbidity/mortality*	
Liver abscess	2 (2%)
Partial thrombosis of IVC	1 (1%)

Of those for which it was reported, 97% of patients had no complications or deaths. Two patients (2%) developed liver abscesses and one patient (1%) developed a partial thrombosis of the IVC. For those reported, there were no patients that failed laparoscopy requiring a second operation. Post-operative complications were not reported in six patients.

### Secondary sources

All nine secondary sources (six review articles, two management guidelines, and one textbook chapter) meeting inclusion criteria commented favorably on the use of DLPW in blunt abdominal trauma patients managed non-operatively [2, 6, 14, 25–29, 31]. Hepatic injury-associated bile peritonitis was the most commonly recommended indication for the procedure, with multiple sources citing resolution of systemic inflammatory response syndrome (SIRS) features including tachycardia and fever, as well as ileus and respiratory failure following washout. In the event that biliperitoneum was confirmed, ERCP was commonly advised as a follow-up for evaluation and intervention of possible ongoing bile leak. Other recommended indications for DLPW included retained hemoperitoneum, hepatic collections, and decompression of abdominal compartment syndrome with a large fluid component. When indicated, most sources preferred delayed laparoscopic washout 2–5 days after initial trauma.

### Quality of evidence

The mean score on the Newcastle–Ottawa Scale across the 10 comparative studies was 6.9 (0.3), with an average of 3.9 stars allocated for the selection domain, 0 for the comparability domain, and 3 for the outcome domain. According to the AHRQ standards, this corresponds with poor quality of evidence. All ten studies received 0 stars in the comparability domain, thus meeting the criteria for poor quality of evidence.

## Discussion

The rise of non-operative management for stable blunt abdominal trauma patients has been followed by an increase in popularity of adjunctive interventions to manage injury patterns and complications that were previously dealt with incidentally during an exploratory laparotomy. Fluid collections, bile leaks, and large hematomas are among these common sequelae that can in turn cause peritonitis, ileus, and a SIRS response which—without appropriate intervention—increase morbidity and prolong hospitalization and rehabilitation [33]. In this scoping review, we surveyed and organized the available evidence that informs the use of DLPW to address such complications.

Regarding quality of evidence, most came in the form poor quality retrospective data including several observational cohort studies and case reports. Almost all included secondary sources that were included, relied on these same studies as the basis for their recommendations. Although one randomized controlled trial was included, it was of small (*n* = 28) sample size and only published in abstract format, precluding critical analysis [10].

The demographics and injury mechanisms of included patients are consistent with epidemiology previously reported in North American blunt trauma [34]. Injury patterns skewed heavily toward medium (Grade III–IV) liver lacerations. This is readily accounted for by the fact that bile peritonitis is one of the primary indications for delayed laparoscopic washout. Surprisingly, there were no splenic injuries explicitly reported. This may be attributable to the low threshold for splenectomy or splenorrhaphy at the time of a delayed laparoscopic exploration (meeting exclusion criteria) or due to pre-existing recommendations against non-operative management of splenic injury in large volume hemoperitoneum [35]. There was a similar paucity of evidence relating to blunt pancreatic, hollow-organ, or vascular injuries.

In this scoping review, indications for DLPW were primarily identified to be clinical features. A large proportion of cases that were taken to the OR were done so on the basis of changes in imaging, thus highlighting the importance of serial and high-acuity monitoring. Importantly, authors reported clinical improvement after DLPW in terms of symptomatology, vital signs, and biochemistry of patients. The hospitalization course for reported patients revealed relatively high transfusion demands with a mean pre-operative transfusion of more than four units of packed red blood cells, which meets most guideline recommendations for operative intervention [29, 30]. In the past, this has been demonstrated as an independent risk factor for surgical intervention in NOM [36]. Additionally, length of stay and post-operative complications were consistent with previously reported experience with blunt liver and abdominal injuries [37–39].

In addition to the management of injuries and complications following NOM, an added benefit of DLPW is the opportunity for abdominal exploration. Laparoscopy has proven to be beneficial in providing a diagnosis for selected trauma patients [28]. Many of the patients who are candidates for DLPW have some element of diagnostic uncertainty arising from abdominal fluid identified on advanced imaging, the nature of which is often assumed. As an illustration, among 13 patients in the DLPW arm of the included RCT, there were 5 missed injuries and 3 patients requiring operative intervention based on said findings.

### Strengths and limitations

A relative strength of our scoping review is the comprehensive and rigorous search methodology, which identified a sizeable body of research from both published and gray literature highlighting key concepts in DLPW. Our screening and selection process were consistent with excellent inter-rater reliability. Most importantly, as a scoping review, we successfully characterized the existing knowledge to establish current rationale for clinical decision making in DLPW as well as identified needs for new research.

Limitations of our methodology include a lack of a pre-registered protocol and exclusion of delayed laparoscopic washout involving additional surgical interventions. The latter of which was felt to be necessary in order to critically evaluate evidence regarding the impact of washout on its own. Additionally, an important weakness in our results is incomplete data for many qualifying patients including baseline demographics, mechanism of injury, operative indications, and post-operative interventions. Oftentimes, this was due to the grouping of demographics for patients treated with NOM with patients receiving immediate surgical intervention in the original studies. Other limitations include a lack of cases involving extra-hepatic injury and overall poor quality of evidence.

### Future directions

By and large, the evidence we report in this study is based on clinicians reacting to adverse clinical changes in patients. Very few authors reported DLPW on an empiric or preventative basis outside of the single RCT. Regarding the overall quality of evidence, studies included were typically categorized as Sacket level III-V (US Preventive Services Task Force level II-2 to III). Thus, our recommendations for future research would be prospective cohort and randomized studies examining empiric use of DLPW in NOM of blunt abdominal trauma as well as any research investigating its use in extra-hepatic injury.

## Conclusions

Existing literature reporting on DLPW after non-operative management of blunt abdominal trauma is limited primarily to retrospective observational data. These studies support the use of DLPW in patients managed non-operatively who go on to develop SIRS features or significant abdominal symptoms. Its use should thus be considered in patients suspected of developing post-trauma intra-abdominal complications. In particular, the use of DLPW in bile leaks and large volume hemoperitoneum appears to appropriately control the systemic inflammatory response, improve clinical symptoms, and possibly reduce length of stay with good mortality outcomes. Despite limited evidence, its use has been incorporated into popular management guidelines. There are substantial gaps in the literature regarding DLPW in this population due to quality and level of evidence. These are opportunities for future prospective and interventional research.

## Data Availability

Not applicable.
